# Remembering history: Autobiographical memory for the COVID‐19 pandemic lockdowns, psychological adjustment, and their relation over time

**DOI:** 10.1111/cdev.14131

**Published:** 2024-08-14

**Authors:** Tirill Fjellhaugen Hjuler, Daniel Lee, Simona Ghetti

**Affiliations:** ^1^ Department of Child and Adolescent Psychiatry Aarhus University Hospital—Psychiatry Aarhus N Denmark; ^2^ University of California, Riverside Riverside California USA; ^3^ Department of Psychology and Center for Mind and Brain University of California, Davis Davis California USA

## Abstract

This longitudinal study examined age‐ and gender‐related differences in autobiographical memory about the COVID‐19 pandemic lockdowns and whether the content of these memories predicted psychological adjustment over time. A sample of 247 students (*M*_age_ = 11.94, range 8–16 years, 51.4% female, 85.4% White) was recruited from public and private schools in Denmark and assessed three times from June 2020 to June 2021. The findings showed that memories weakened over time in detail and emotional valence. Additionally, psychological well‐being decreased over time, with adolescent females faring the worst. Critically, memories including higher levels of negative affect and factual information about COVID‐19 and the lockdown predicted worse psychological well‐being over time, underscoring aspects of autobiographical memory that might help attenuate the negative consequences of the lockdown.

**Abbreviations**
AMQ: Autobiographical Memory Questionnaire
NE: negative emotion
PM: positive emotion
SMFQ: Short Mood and Feelings Questionnaire
WHO‐5: World Health Organization Well‐Being Index

## INTRODUCTION

The COVID‐19 pandemic and the measures to prevent contagion caused increased anxiety and extensive disruptions in children's and adolescents' everyday social lives (Orben et al., 2020; Varma et al., 2021). The fear of contagion in the community and in response to the news may have altered children's and adolescents' perceptions of safety and increased anxiety (Imran et al., 2020; Pfefferbaum, 2021). Moreover, the unusual everyday life during lockdowns may have reduced the opportunity for the experience of distinctive shared life events (e.g., Bluck, 2003; Habermas, 2012; Pillemer & Kuwabara, 2012), potentially affecting the very ability to reminisce about and remember the past (Brown, 2021). This is important because many argue that autobiographical memory provides a foundation for emotional well‐being (e.g., Banks & Salmon, 2013; Fivush & Baker‐Ward, 2005). Since the onset of COVID‐19, studies have documented acute transient stress reactions in children and adolescents (e.g., Meade, 2021; Varma et al., 2021). However, most existing studies were conducted in the early phases of the pandemic (Jones et al., 2021; Meherali et al., 2021), whereas the pandemic has continued to affect everyday life over an extensive period of time. Moreover, studies suggest that prolonged restrictions had detrimental effects on mental health (e.g., Orben et al., 2020) Therefore, longitudinal studies are needed to determine cumulative or long‐term effects of the pandemic and to investigate how children's and adolescents' autobiographical memories and mental health may be affected. We seek to contribute to this literature by examining longitudinal change in the content of autobiographical memories about the COVID‐19 lockdown periods as a function of age and gender. Moreover, we sought to investigate whether certain aspects of autobiographical memory content predicted worsening of well‐being during this time period. Although we are unable to fully evaluate the long‐term consequences of the COVID‐19 lockdowns on psychological adjustment some of which are still ongoing, our study provides new insight on the changes in autobiographical content and well‐being across separate lockdown periods and returns to school occurring in Denmark between Spring 2020 and Spring 2021. A further discussion of the Danish context is included in a later section.

### Autobiographical memory for events of historical relevance

Autobiographical memory is the ability to remember and relive personal experiences and to place them into a personal life‐historical context (Berntsen & Rubin, 2012). Memories of personal past events are essential for an individual's sense of self because they capture self‐defining aspects of one's life, support meaning‐making of one's past (Nelson, 1993), and provide a sense of continuity over time (Berntsen, 2009; e.g., Conway, 2005; Thomsen, 2015; Thomsen et al., 2011). According to Brown et al. (2012), events of historical relevance may shape individuals' autobiographical memories by providing temporal milestones and thematic labelling, often referred to as “historically defined autobiographical periods”. However, Brown (2021) claimed that the changes in everyday life during COVID‐19 may shape individuals' autobiographical memories differently compared to other historical periods and events. Specifically, Brown (2021) predicted an increase in personal memories for the onset of COVID‐19, which he termed a “COVID bump”, and a decrease in accessibility and recollection of details in personal memories from lockdown periods compared to other more stable periods, termed a “lockdown dip”. Brown's predictions are consistent with theories highlighting that individuals typically remember salient, specific events that stand out from everyday repeated and schematized events (Barsalou, 1988; Neisser, 1986). During lockdowns, social distancing and isolation restricted individuals' opportunities for experiencing novel, memorable events. From this perspective, we might expect that children and adolescents would remember well their initial discovery of COVID‐19 or early phases of the lockdown, but their memories for the events would decline over time as each new day likely brought minimal opportunities for new experiences. The quality of autobiographical recollections is often measured in terms of episodic content (i.e., the extent to which memories include specific details) and semantic content (i.e., factual information) (Levine et al., 2002). Thus, we should expect stronger episodic content earlier compared to later in the pandemic.

According to Levine et al. (2002), episodic autobiographical content is foundational to retain precise and vivid representations of personally experienced events, whereas semantic autobiographical content supports coherence of self‐knowledge and the feeling of identity over time. Similarly, Conway and Pleydell‐Pearce (2000) proposed a hierarchical model of autobiographical memory, in which a person's self‐knowledge connects with several themes reflecting specific lifetime periods or certain events. Relatedly, the emotional aspects of autobiographical memories add further to the complexity. In general, autobiographical memory is biased towards emotionally positive events, meaning that positive events are more frequently and more easily recalled compared to emotionally negative events (Berntsen & Rubin, 2012). Berntsen and Rubin (2012) argue that this may be explained by humans' strive towards maintaining a positive view of oneself and others, which is essential for the ability to engage in social relationships. However, in the context of COVID‐19 and the lockdown, this function may be attenuated or manifested differently as people's lives were confined resulting in limited self‐determination and social interaction. Hence, the memories may be more likely to capture the negative impact of lockdowns, thus reflecting increased negative emotional content. In addition, some children and adolescents might struggle to maintain a positive coherent self‐knowledge over time, which may be manifested with a tendency to integrate more general factual information about COVID‐19 than information about personal events.

### Development of autobiographical memory from middle childhood to adolescence

Previous studies have documented developmental changes in autobiographical memory construction (Fivush et al., 2012; Fivush & Baker‐Ward, 2005). Autobiographical memory develops gradually across childhood and adolescence (Bauer, 2007; Fivush, 2011; Wang, 2013) in tandem with changes in several important factors including meaning‐making in personal narratives (Bohanek & Fivush, 2010; Koch & Wang, 2022). Even though emerging autobiographical memory capabilities have been observed in young children, such as preschoolers (e.g., Reese & Newcombe, 2007; Ross et al., 2019), studies indicate that children's remembering of personal events becomes more robust by the time they reach school age, when they start to remember personally experienced events in more detail over lengthy delays (Bauer, 2007; Harley & Reese, 1999). During this time, autobiographical memories become more complex, coherent, and personally integrated (Habermas & Bluck, 2000). Importantly, research suggests that the capability to construct and integrate memories from past experiences into a narrative identity emerges during adolescence (Fivush, 2011). Similarly, studies indicate that the ability to provide coherent life stories develops in parallel with life script normativity (Bohn & Berntsen, 2008), and it is not until about 12 years of age that adolescents start to consistently link individual memories of experienced events together to form a genuine narrative (Habermas & de Silveria, 2008). Thus, older adolescents may integrate and thereby reflect more broadly on their memories from the lockdown periods compared to their younger peers.

Gender is also an essential factor in the construction of autobiographical memories (Fivush, 2012). In a study of 13‐ to 16‐year‐olds, Fivush et al. (2012) found that females' personal memories were overall more elaborated, coherent, and reflective. Moreover, females' autobiographical memories are typically longer, more detailed, and include more emotional detail compared to males' (Buckner & Fivush, 1998; Niedźwieńska, 2003). Therefore, adolescent girls may be particularly likely to report elaborated memories on how different aspects of COVID‐19 affected their lives and emotional well‐being compared to boys. Habermas (2015) argued that personal narratives serve two major functions, namely communication of the experienced events and their evaluation. Hence, autobiographical memories likely capture children's and adolescents' experiences and responses to the COVID‐19 lockdowns, and the way they are expressed likely differ across age and gender with regards to e.g., details and emotional evaluation. Thus, in this study, we considered a combined examination of autobiographical memories and mental health essential in order to provide a more comprehensive understanding of the impact on children's and adolescents' lives and well‐being during lockdowns.

### Relations between autobiographical memory and children's and adolescents' psychological well‐being

Adolescents' mental health symptoms have been on the rise over the last decade (Burstein et al., 2019; Keyes et al., 2019; Marquez & Long, 2021). Adolescent females tend to report lower levels of well‐being compared to males, and the prevalence of life satisfaction has been shown to decline in girls from age 11 to age 15 (e.g., Gregory et al., 2021). Moreover, the onset of many psychiatric disorders can be traced to childhood or adolescence. For example, half of all lifetime cases of psychiatric disorders have their onset by age 14 and three fourth by age 24 (Kessler et al., 2005). Thus, psychological well‐being should be promoted during childhood and adolescence to reduce the risk of mental disorders. Positive mental health and well‐being are typically facilitated through positive engagement and socialization with friends, family, teachers, and the community (e.g., Bethell et al., 2019). A review by Orben et al. (2020) illustrates how face‐to‐face peer interaction is a vital aspect of adolescent development, and how limited social interaction may negatively affect the brain and behavior. However, as COVID‐19 infection rates increased, social distancing and reduced peer face‐to‐face interaction were encouraged, if not mandated. Hence, as documented by previous studies (e.g., Loades et al., 2020; Meherali et al., 2021; Orben et al., 2020), the combination of typical stressors emerging in adolescence and COVID19‐related stressors may have led to robust decrease in adolescents' mental health. For instance, findings suggest that children and adolescents were even more likely to experience loneliness, anxiety and depression compared to adults during lockdowns (e.g., Loades et al., 2020; Meherali et al., 2021), which may place them at increased risk of long‐term consequences on mental health (e.g., Meherali et al., 2021; Orben et al., 2020).

Psychological well‐being and autobiographical memory are closely intertwined. Autobiographical memory supports emotion regulation (Banks & Salmon, 2013; McLean & Lilgendahl, 2008) and psychological well‐being (McLean et al., 2010; Watson & Berntsen, 2015). Studies have documented that autobiographical narratives that include descriptions of personal growth and positive aspects of the self, predict positive psychological well‐being (Lilgendahl & McAdams, 2011; Pals, 2006). In remembering and constructing personal narratives of past events, individuals create meaning and achieve a more profound understanding of previous experiences (Fivush et al., 1996) and self‐understanding (Conway & Pleydell‐Pearce, 2000). COVID‐19 has had a profound impact on children's and adolescent's everyday lives; for example, the periods of lockdown restricted social interactions with peers, which are deemed particularly vital in adolescence (Baumeister & Leary, 1995). Therefore, we expected that children and adolescents who included more negative content in their memories and more factual COVID‐19‐related information (as opposed to descriptions of personal experiences) would exhibit lower levels of well‐being.

### Research in context: COVID‐19 lockdowns in Denmark from spring 2020 to summer 2021

The COVID‐19 pandemic has had and continues to have a tremendous worldwide impact on people's lives. Children and adolescents experienced a prolonged period of uncertainty with disruptions in social routines due to policies fostering reduced contact among people. UNESCO (2021) reported that school closures affected 1.6 billion students in 190 countries during Spring 2020. In Denmark, where this study was conducted, the first school closure was mandated on March 11th, 2020. School closure lasted until April 17th, 2020 for younger students (Preschool to 5th grade), and persisted until May 18th, 2020 for older students (6th to 9th graders). As in other countries, the numbers of COVID‐19 infections increased drastically during fall 2020, resulting in an additional period of school closure from December 17th until May 6th, 2021. In other words, children's and adolescents' social life was disrupted for 5 months during the second school closure, a sizeable percentage of their lived lives. Moreover, studies suggest that children and adolescents perceive time as passing more slowly than adults do, which might be due to neurocognitive changes and memory aspects (Bejan, 2019). Thus, half a year lockdown is an extensive period in children and adolescents' lives.

In addition to school closures, the Danish lockdowns included more extensive government‐enforced lockdown measures, including closure of leisure activities (e.g., movie theater, sports), social distancing, ban of gatherings, work‐from‐home orders and mask mandates. To date, two studies on the effect of the pandemic on adults' memory retrieval (Cole et al., 2023; Öner et al., 2023) suggest that COVID‐regulations at a national level may be particularly likely to affect memory content. In both studies, a stringency index was used as a measure of the governmental response to the pandemic at a national level, which consisted of a composite score across several restrictions, developed by the University of Oxford (for details, see Hale et al. (2021)). The scores range from 0 to 100, with higher scores including stricter governmental response. According to Cole et al. (2023), the Danish stringency index corresponded to 43.90 (as a comparison the score was, 37.69 for the USA and 69.83 for China). The mean stringency index between all 14 countries included in the study, was 53.10. The studies revealed associations between the level of stringency and the content of autobiographical memories such that themes in narratives in countries with high severity and low stringency (e.g., USA) were primarily centered around deaths as opposed to in countries with low severity but high stringency (e.g., China). However, autobiographical memories from people in countries with high stringency index were higher in negative emotional valence compared to countries with less strict regulations (Cole et al., 2023; Öner et al., 2023). Still, no previous study has measured the longitudinal change of the content of children's and adolescents' autobiographical memories during the pandemic. In addition, even though studies have measured children's and adolescents' mental health over time during the pandemic, there is a lack of knowledge on *if and how* autobiographical memories about the lockdown differ between age and gender, and whether the memory content may affect mental health during the same period. Given the extraordinary importance of this period worldwide, and the extended period of isolation, we deemed it important to examine long‐term effects on children's and adolescent's mental health as well as aspects of autobiographical memories related to the period, and how their content might affect mental health over time.

### The present study

The present study investigated age‐ and gender‐related differences in autobiographical memories from COVID‐19 lockdowns in 8‐ to 16‐year‐olds. These age groups were selected because they cover an extensive developmental period during which autobiographical memory develop substantially (Fivush, 2012) and during which psychiatric disorders begin to emerge (Kessler et al., 2005).

We assessed autobiographical memories and mental health at three time‐points starting in June 2020 immediately after the first lockdown ended, in January 2021 during the second lockdown, and in June 2021 following the end of the second lockdown. At all three time‐points, we assessed autobiographical memories from the lockdown, psychological well‐being and depressive symptoms. The content included in the autobiographical narratives was coded for Episodicity (i.e., level of episodic detail for personally experienced events), corona semanticity (i.e., factual information about COVID‐19), and emotional tone (i.e., positive and negative affective content). The coded material was used to obtain objective measures of the memory content and to compare age‐ and gender differences.

We expected an overall increase in negative affect and a decrease in Episodicity and corona semanticity over time due to the overall negative impact of the lockdowns and decreased opportunities for experiencing new, memorable events (Brown, 2021), and we expected that the changes would be more pronounced among adolescent females because of their increased proneness to negatively affected psychological well‐being. We also predicted that the overall mental health would decrease over time (Meherali et al., 2021; Orben et al., 2020) and that adolescent females would display the worse mental health scores (Gregory et al., 2021). Finally, we expected that the content of autobiographical memories would prospectively predict psychological well‐being such that more negative and more semanticized autobiographical memories would predict worse mental health adjustment, including depressive symptoms and lower levels of psychological well‐being.

## METHOD

### Participants

Participants included 247 students (120 males, 127 females) enrolled in grade second (*N* = 71, *M*_age_ = 8.52, SD = 0.56), fifth (*N* = 74, *M*_age_ = 11.46, SD = 0.53) and eight (*N* = 102, *M*_age_ = 14.68, SD = 0.59). The sample mean age was 11.94 years (SD = 2.61), range 8–16 years. The mean age for males was 12.03 years (SD = 2.57) and females was 11.87 years (SD = 2.66). Data were collected in June 2020 (Time 1). Participants were recruited from five schools (three public and two private schools) in the region of Central Jutland, Denmark. Income information was not collected from the school setting, but these schools serve economically diverse populations of children (2 schools served parents with an overall income below the average population, 1 school served parents with an overall income above the average population, and 2 schools served parents with an overall average income). Children's race was 85.4% White, 2% Black, 7.7% Asian and 4.9% Mixed. At Time 2 (January 2021), 158 (76 males, 82 females) of the 247 students participated from third (*N* = 24), sixth (*N* = 57) and ninth (*N* = 77) grade. At Time 3 (June 2021) 202 (97 males, 105 females) of the 247 students participated from third (*N* = 54), sixth (*N* = 64) and ninth (*N* = 84) grade. Every class received snacks for participating. The study was approved by the local ethics committee, and prior to participation, written and informed consent was obtained by both the parents and the participating children.

### Materials

#### Autobiographical Memory Questionnaire

The Autobiographical Memory Questionnaire (AMQ) was designed to assess the content of children and adolescents' memories from the lockdown periods (e.g., episodic detail, semantic features, emotional valence, and subjective experience). First, participants were asked to describe the most memorable personal event from the lockdown period. Instructions emphasized that there was no right or wrong answers and that we were interested in their own personal experiences from the period. Specifically, the instructions stated: “Please write down as many details as possible about a memory from your personal life during the COVID‐19 lockdown.” After providing their narrative, participants were asked to rate several aspects of their memory using Likert scales ranging from 1 = not at all to 5 = Very much. Specifically, participants assessed the extent to which their memory for the event was: (a) Positive; (b) Negative; (c) Important to me; (d) Stressful; (e) “Hard to think about”. In addition, using a 5‐point Likert scale, they answered the questions: “How well do you remember this life event?” as (a) Clearly (from not clearly at all to very clearly); (b) Detailed (from not detailed at all to very detailed); and “How does the memory make you feel?” (all emotions rated from ‘not at all’ to ‘very much’): (a) Happy; (b) Satisfied; (c) Calm; (d) Sad; (e) Angry; (f) Worried; and “How often have you thought about this memory?” (from never to very often), and (V) “How often have you talked about this memory?” (from never to very often). Finally, participants were asked to report whether they could see the event primarily from a first person perspective (‘I’) or a third person perspective (‘he’/'she’) if they could visualize the specific personal event in their mind. The Procedure section provides a thorough description of how we introduced and explained the AMQ to our participants. We note that the results regarding participants' ratings over time are not reported in the main article, but they can be found in the Supporting Information. The results concerning the content of autobiographical memory reported in the main article are based entirely on the coders' ratings of the participants' written narratives (see section on Memory Coding for details).

#### World Health Organization Well‐Being Index (Topp et al., 2015)

The World Health Organization Well‐Being Index (WHO‐5; Topp et al., 2015) is among the most widely used assessment tool for subjective psychological well‐being over time (Topp et al., 2015). This measure is validated for children aged 9 and above (Allgaier et al., 2012). The questionnaire consists of 5 simple questions (e.g., ‘I have felt cheerful and in good spirits’) requiring respondents to indicate how accurately the statement described their feelings over the past 2 weeks, on a 5‐point scale ranging from 1 = Never, to 5 = All the time. The score is based on the total raw sum derived from the respondent's answers (ranging from 0 to 25) which is multiplied by 4 to provide the final score, with 0 representing the worst possible well‐being and 100 representing the best possible well‐being. Lower scores indicate poorer well‐being, where scores below 50 indicate a possible risk of depression (Topp et al., 2015). This questionnaire was administered exactly as indicated by guidelines for the assessment of current psychological state. In addition, we asked participants to rate their well‐being during the lockdown when we did not have yet access to the students. For that retrospective assessment, we replaced “in the past 2 weeks” with “during the lockdown”.

#### Short Mood and Feelings Questionnaire (Angold et al., 1995)

The Short Mood and Feelings Questionnaire (SMFQ; Angold et al., 1995) measures core symptoms of depression in children and adolescents aged 6 to 17 years old. Specifically, the SMFQ includes 13 statements assessing subjective affective and cognitive symptoms of depression that the respondents have experienced over the previous 2 weeks. Participants are asked to rate each statement (e.g., ‘I cried a lot’/‘I felt miserable or unhappy’) on a 3‐point Likert scale with 0 = not true, 1 = sometimes true; 2 = true. The final score is calculated by summing the point values on each item response, ranging from 0 to 26 where higher scores indicate higher prevalence of depressive symptoms. According to Angold et al. (1995) scores over 8 suggest the presence of significant depressive symptoms. As for the WHO‐5, we also administered this instrument to assess symptoms during the lockdown. Thus, children completed the scale again and for each question, the expression “in the past 2 weeks” was replaced with “during the lockdown.”

### Procedure

**FIGURE 1:**
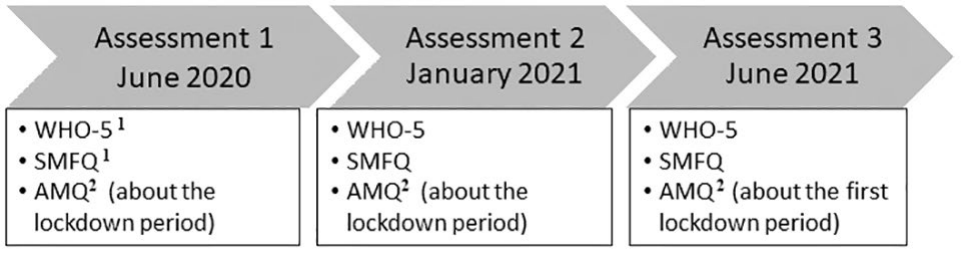
A schematic illustration of our assessment schedule. ^1^During Assessment 1, these measures were completed twice, once focusing retrospectively on the lockdown period and once focusing on current psychological state; the other assessments only required reports about current psychological state. ^2^Assessment 3 occurred after two lockdown periods, therefore distinct memories for the first and second lockdown were elicited.

We scheduled data collection in the schools in agreement with the administrators of each of the 5 participating schools. For feasibility reasons, we developed our methods such that they could be administered as a survey to be distributed and completed independently during classroom time. The first author (TFH) trained the students carefully on how to respond to the questions included in the survey by providing a wealth of examples of everyday life events not associated with COVID‐19 which were rated as a group in the classroom by using a Likert scale drawn on the blackboard. For example, TFH stated: “Think back about the last time you went to the movies. Now, let's rate it together. How positive is your memory of this event?” [‘Going to the movies’ was used as an example because the movies were all closed during the Danish lockdowns. Thus, ‘going to the movies’ would likely not be a memory recalled from lockdown from any participant when completing the survey following training]. Moreover, TFH particularly explained the difference between 1st and 3rd person perspective. The students were encouraged to raise their hands to ask clarification questions about the content of each item they were going to be asked about or how to use the Likert scales. Participants were not provided information about the specific goals of the study or hypotheses pursued. TFH was physically present in the classroom during data collection for Time 1 and Time 3, when children were tested in school and stayed during data collection to address any question students may have had during participation. Data collection for Time 2 was completed during remote classroom time during the second Danish lockdown. The teachers were provided with detailed written instructions to guide them through the administration of the survey. Importantly, the participants were instructed that there was no right or wrong way to write about and rate their memories, as we were interested in *their own personal* experiences (Figure 1).

#### Time 1 assessment

Participants completed the Danish version of the WHO‐5 (Topp et al., 2015) and the SMFQ (Angold et al., 1995), assessing well‐being and depressive symptoms respectively. We asked participants to complete these measures twice, once assessing their well‐being retrospectively in relation to the time of the first lockdown, and a second time in relation to their current state. This was a compromise given that we did not have the chance to start our study during the first lockdown. Finally, participants were asked to provide an autobiographical memory from the first lockdown period.

#### Time 2 assessment

The assessment included the same measures included in Time 1 assessment except that we only asked participants to complete the measures in relation to their psychological current state (WHO‐5 and SMFQ). Also, because the Time 2 assessment took place *during* the second lockdown the wording of the AMQ read “Please write down as many details as possible about a memory from your personal life during the COVID‐19 period, which began with the first lockdown in March 2020.” Similar to the Time 1 assessment, participants were subsequently asked to rate specific aspects of their memory using the equivalent Likert scales.

#### Time 3 assessment

The assessment included the same measures included in Time 1 Assessment regarding the current psychological state (WHO‐5 and SMFQ) and the AMQ, except that at Time 3, we asked for a memory from both the first lockdown, the second lockdown, a personal memory not related to lockdowns and a future projection. (However, the memory from the second lockdown, the personal memory and the future projection is not examined in this report). Once again, the participants were asked to provide subjective ratings about their memories.

#### Memory coding

In order to obtain objective measures of the content of the autobiographical memories, these were coded for (1) Episodicity, (2) Corona Semanticity, (3) Emotional Valence, and (4) theme by nine independent raters blinded to the hypotheses and participants' age and gender. The list of themes and their frequency are reported in Supporting Infomation.

##### Episodicity

The Episodicity of each memory was coded using a 4‐point scale (0–3) adapted from a scale developed by Piolino et al. (2003) and other adaptations successfully used in studies with children (e.g., Coughlin et al., 2014, 2019; Piolino et al., 2007). A code of 0 was given when no episodic content was provided in the memory reported. A code of 1 was given when participants only reported a vague event that was repeated or continuous with little or no detail of time or space but referring to a specific instance of an event (e.g., “I wanted to go there”, “I gamed”). A code of 2 was given when the participant reported a specific event with only one or few contextual details from the categories ‘time’, ‘space’, ‘imagery’, ‘thoughts’ and ‘emotions’ (e.g., “A Friday during lockdown I attended an online exam”, “I felt scared when the schools re‐opened and we had to go back”). Finally, a code of 3 was given when the participant reported a specific event with several contextual details from the categories ‘time’, ‘space’, ‘imagery’, ‘thoughts’ and ‘emotions’ (e.g., “A Friday during lockdown I attended an online exam and I felt very nervous so I decided to call my friend in the morning”). Two raters (rater ‘A’ and rater ‘B’) were trained by the first author based on a detailed coding manual until they sufficiently agreed on issues discussed during training. Subsequently, both raters coded all the memories. The interrater agreement was high, 92.3%, *κ* = .867, (8‐ to 9‐year‐olds' memories: 96.0%, *κ* = .939; 11‐ to 12‐year‐olds' memories: 92.8%, *κ* = .858; 15‐ to 16‐year‐olds' memories: 89%, *κ* = .760). The coding used for the analyses were the mean scores calculated from both raters' coding.

##### Corona Semanticity

The corona semanticity for each memory was also coded using a 4‐point scale (0–3) adapted from Levine et al. (2002) A code of 0 was given when the participant did not include any semantic information about corona or the lockdowns. That is, the participant only included episodic information in the memory and/or semantic information not related to corona or the lockdowns. A code of 1 was given when the participant solely mentioned the words ‘corona’ or ‘lockdown’, but with no further independent semantic information about the period (e.g., “We were in lockdown for a period.”). A code of 2 was given when the participant reported an overall general semantic knowledge related to corona or the lockdown, but with no further specific details (e.g., “During the lockdown, we were told to stay at home”, “The schools were closed down”). And a code of 3 was given when the participant reported general semantic knowledge including specific details about corona or the lockdown (e.g., “During the lockdown, everyone was told to stay at home and to wear a face mask and use hand sanitizer when going somewhere”). Two raters (rater ‘C’ and rater ‘D’) were trained by the first author based on a detailed coding manual until they sufficiently agreed on issues discussed during training. Subsequently, both raters coded all memories. The interrater agreement was high, 94.8%, *κ* = .858, (8‐ to 9‐year‐olds' memories: 97.6%, *κ* = .939; 11‐ to 12‐year‐olds' memories: 90.0%, *κ* = .809; 15‐ to 16‐year‐olds' memories: 95.1%, *κ* = .922). The coding used for the analyses were the mean scores calculated from both raters' coding.

##### Emotional Valence

The emotional valence of each memory was coded as two separate dimensions (positive and negative) on a 3‐point scale with 0 = no positive/negative emotions or evaluations, 1 = some positive/negative emotions or evaluations, 2 = many and overweight of positive/negative emotions or evaluations, based on Thomsen and Vedel (2019). Two raters (rater ‘E’ and rater ‘F’) were trained by the first author on the basis of a detailed coding manual. Coders were asked to start out by reading each narrative and highlighting words and sentences that referred to positive/negative emotions (e.g., sad, happy, boring), evaluations, (e.g., it was bad, it went well), qualities (e.g., anxious, brave, wrong,) and verbs (e.g., enjoy, cry). Subsequently, the highlights were marked by either PM (positive emotion) or NE (negative emotion). When all the highlights had been marked, the memory was coded for PE and NE as two separate codes in a coding scheme. The first author provided ‘training memories’ based on the original data, which were used for training until the raters sufficiently agreed on issues raised during training. Subsequently, both raters coded all memories. The interrater agreement was high, 97.7%, *κ* = .926 (PE = 96.4%, *κ* = .923, NE = 99.0%, *κ* = .923), (8‐ to 9‐year‐olds' memories: 98.0%, *κ* = .947; 11‐ to 12‐year‐olds' memories: 98.0%, *κ* = .951; 15‐ to 16‐year‐olds' memories: 95.1%, *κ* = .896). The coding used for the analyses were the mean scores calculated from both raters' coding.

### Analytical approach

Multilevel modeling analyses were employed to assess intraindividual change as well as average change across participants in autobiographical memory content over time and psychological well‐being and depressive symptoms. We used multilevel modeling techniques due to their sensitivity to differences in groups and because of its ability to account for missing data over the three waves (e.g., Bryk & Raudenbush, 1987; Tasca & Gallop, 2009). First, we examined psychological adjustment over time (i.e., WHO‐5 and SMFQ) as a function of age, and gender, and assessment time; interaction effects between these variables were also tested. Second, we examined the content of memory by assessing subjective and objective memory measures as a function of age, gender, and time assessment. Please note, that the analyses on the memory content presented in the main article, are all based on the objective ratings (i.e., memory codings). Finally, we investigated whether unique memory content variables predicted psychological adjustment during the COVID‐19 lockdowns measured from June 2020 to June 2021. Specifically, we examined Emotional Valence [Negativity], Episodicity and Corona Semanticity. Age, gender, and time were also included in the models.

## RESULTS

### Preliminary analyses

First, we assessed the internal consistency of the WHO‐5 index and the SMFQ measure over time. The measures demonstrated high internal consistency reliability across the three waves of data collection with an average WHO‐5 of *α* = .76 and SMFQ of *α* = .87 (each measure demonstrated the following internal consistency scores: WHO‐5 index: T1 lockdown *α* = .78; T1 present *α* = .71; T2 *α* = .76; T3 *α* = .79; SMFQ; T1 lockdown *α* = .85; T1 present *α* = .85; T2 *α* = .88; T3 *α* = .89). Additional analyses examining scale reliability in each age group separately was conducted with the WHO‐5 because it is not validated for our youngest group. We found that reliability was acceptable across time‐points for each age group (Average alpha, .71 in 8‐ to 9‐year‐olds; .74 in 11‐ to 12‐year‐olds and .78 in 15‐ to 16‐year‐olds). Moreover, we examined the construct validity of the WHO‐5 by conducting correlation analyses between WHO‐5 and SMFQ across each point of assessment which showed a strong or moderate negative correlation (T1 lockdown on average, *r* = −.60, *p* < .001, (8‐ to 9‐year‐olds, *r* = −.60, *p* < .001; 11‐ to 12‐year‐olds, *r* = −.68, *p* < .001; 15‐ to 16‐year‐olds, *r* = −.75, *p* < .001); T1 present on average, *r* = −.67, *p* < .001, (8‐ to 9‐year‐olds, *r* = −.50, *p* < .001; 11‐ to 12‐year‐olds, *r* = −.64, *p* < .001; 15‐ to 16‐year‐olds, *r* = −.68, *p* < .001), T2 on average, *r* = −.70, *p* < .001, (8‐ to 9‐year‐olds, *r* = −.71, *p* < 001; 11‐ to 12‐year‐olds, *r* = −.76, *p* < .001; 15‐ to 16‐year‐olds, *r* = .67, *p* < .001), and T3 on average, *r* = −.63, *p* < .001, (8‐ to 9‐year‐olds, *r* = −.60, *p* < .001; 11‐ to 12‐year‐olds, *r* = −.66, *p* < .001; 15‐ to 16‐year‐olds, *r* = −.63, *p* < .001)).

Second, chi square tests of independence were conducted to compare frequencies of retained versus lost participants over time as a function of gender, *χ* ^2^(1, 247) = 0.15, *p* = .69 and age, *χ* ^2^(1, 247) = 3.55, *p* = .17. A Welch's two‐sample *t*‐test showed no significant differences between the retained and lost participants in the WHO‐5 scores, *t*(173.86) = 0.22, *p* = .82, or in the SMFQ scores, *t*(176.48) = 1.51, *p* = .13.

### Memory content over time—Objective measures

Multilevel models were utilized to assess changes in the objective content of the memories (i.e., coded from participants' narratives). First we used a linear mixed model to assess the overall change in memory content over time. Second, multilevel models assessed changes in memory content as a function of age, gender, and time. Specifically, we analyzed changes over time in the memories' Episodicity, Corona Semanticity and Emotional tone.

The analysis of changes in Episodicity revealed an overall strong decrease over time, *β* = −0.25, *t* = −6.16, *p* < .001 (i.e., indicating .26 of a standard deviation change for each year). When we added age and gender in addition to time in the model, we found an effect of age with greater levels of Episodicity in the oldest compared to youngest age group (*β* = 0.89, *t* = 2.83, *p* < .01) and a marginal effect of time (*β* = −0.23, *t* = −1.93, *p* = .05). We found no effect of gender (*β* = 0.08, *t* = 0.23, *p* = .82). Interactive effects were not statistically significant (*β* = −0.11, *t* = −0.54, *p* = .59).

**FIGURE 2:**
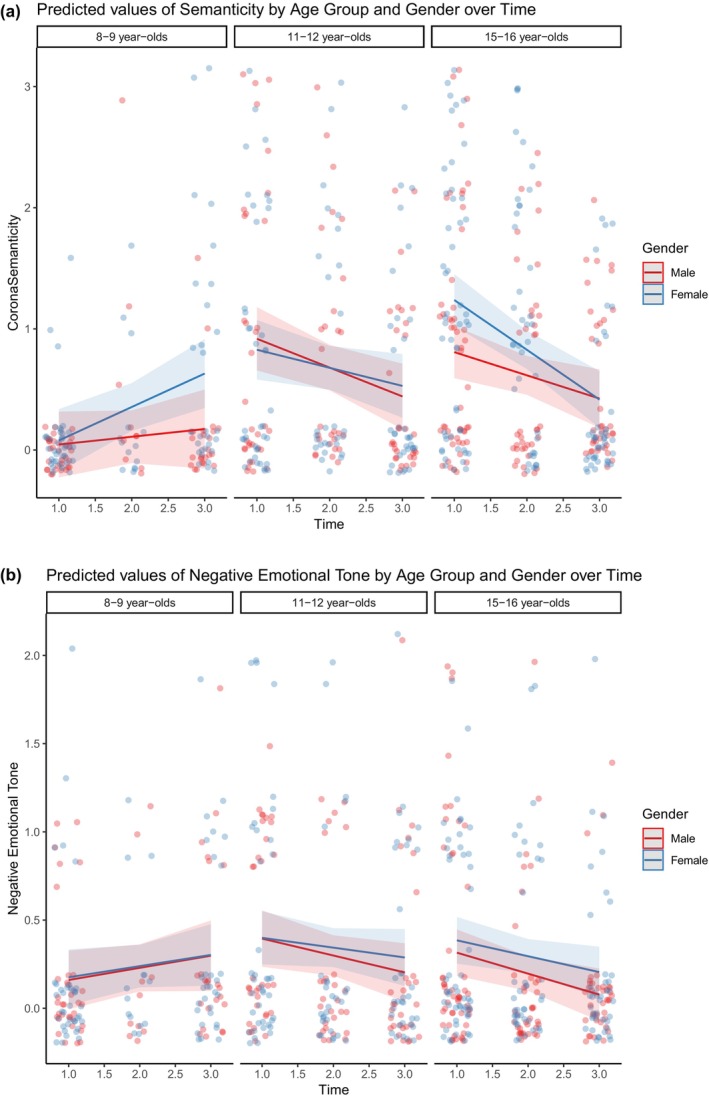
Predicted values of Corona Semanticity and Negative Emotional Tone over time with age and gender effects. Note that jitter is added to the data points to be able to show the individual males and females. The time‐points on the *x*‐axis are displayed in steps of .5.

As for Corona Semanticity, we also found an overall decrease over time (*β* = −0.14, *t* = −3.28, *p* < .01) in the model examining the effect of time alone. When we analyzed the Corona Semanticity as a function of age, gender, and time, we found a main effect of age, such that the younger age group in this study reported lower levels of semantic information about COVID‐19 than did both the middle age group (*β* = 1.33, *t* = 3.85, *p* < .001) and the oldest age group (*β* = 1.15, *t* = 3.62, *p* < .001). The examination of interaction effects with our time variable revealed a significant interaction between age, gender, and time (*β* = 4.89, *t* = 2.35, *p* < .05). The two older groups showed declines in semanticity over time, with the middle age group declining overall (*β* = 3.41, *t* = 2.12, *p* < .05), and decline more pronounced in girls in the older group. In other words, older female students included higher levels of corona semanticity at the beginning at the lockdown compared to younger age groups and to males, but they then displayed a steeper decrease in corona semanticity over time (see Figure 2, panel A).

Our results also showed an overall decrease in the Positive Emotional Tone over time, *β* = −0.09, *t* = −2.01, *p* < .05. The analysis examining Positive Emotional Tone as a function of age, gender and time, revealed an effect of age, such that the older age group reported greater levels of Positive Emotional Tone (*β* = 0.81, *t* = 2.43, *p* < .05) compared to the middle and the younger age group. Similarly, females reported greater levels of Positive Emotional Tone in their memories (*β* = 0.76, *t* = 2.13, *p* < .05). No significant interactive effects with time were found (*β*s ≤ 0.6, *t* ≤ 0.45, *p* ≥ .15).

Similarly, and somewhat surprisingly, we also found an overall decrease in the Negative Emotional Tone over time (*β* = −0.10, *t* = −2.22, *p* < .05). When we examined the full model including age, gender and time, we found that the middle age group reported memories with greater Negative Emotional Tone (*β* = 0.76, *t* = −2.1, *p* < .05). In addition, the results revealed a significant interaction between age and time, such that a more pronounced decline in Negative Emotional Tone was found in the oldest age group (*β* = −0.36, *t* = −2.26, *p* < .05) (see Figure 2, panel B).

### Psychological adjustment over time

**FIGURE 3:**
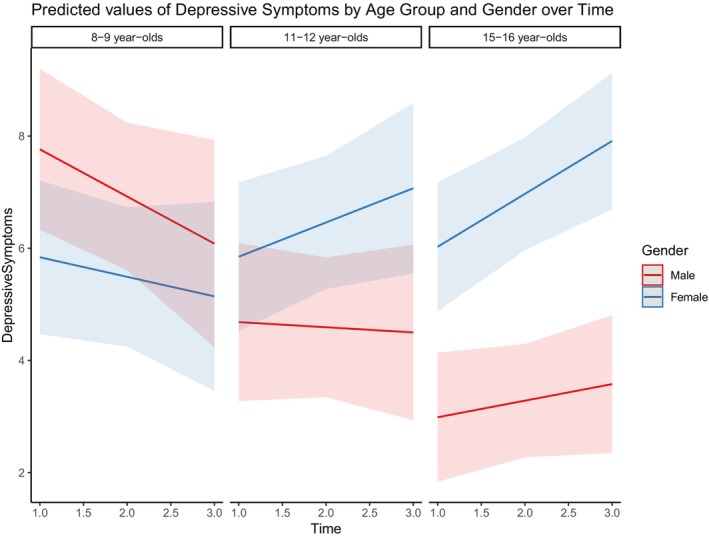
Depressive symptoms over time divided by age and gender.

We hypothesized a decrease in mental health over time. A multilevel linear model suggests that the overall psychological well‐being measured by the WHO‐5 index decreased over time (*β* = −1.56, *t* = −3.64, *p* < .001). Older females and especially the 11‐ to 12‐year‐old females, exhibited the overall lower levels of well‐being based on the WHO‐5 index over time (*β* = −4.76, *t* = −3.64, *p* < .05). Although less pronounced, 15‐ to 16‐year‐old females' well‐being also decreased over time (*β* = −4.05, *t* = −1.9, *p* = .06). Additionally, the model assessing depressive symptoms measured by SMFQ showed an overall increase in depressive symptoms over time, although less pronounced than well‐being (*β* = 0.04, *t* = 2.06, *p* < .05). The 15‐ to 16‐year‐old adolescent females exhibited greater levels of depressive symptoms overall (*β* = 3.56, *t* = 2.22, *p* < .05) and all though not being significant, their level of depressive symptoms seemed to increase over time (*β* = 0.86, *t* = 1.67, *p* = .09). Changes in depressive symptoms measured by the SMFQ‐index over time, divided by age and gender is illustrated in Figure 3.

### Memory content predicting psychological adjustment

Finally, we tested the longitudinal relation between the content of memories and psychological adjustment. To do so, we implemented the same model used to predict psychological adjustment over time while accounting for gender and age and added Episodicity, corona semanticity, and negativity as predictors including their interaction with time to analyze the trajectory of these relationships. Overall, the analyses revealed that memories with higher levels of negative content (*β* = 1.84, *t* = 2.69, *p* < .01) and lower levels of Corona Semanticity (*β* = −1.84, *t* = −2.65, *p* < .01) predicted greater psychological well‐being measured by the WHO‐5 index over time. We tested an additional model to include not only the main effects of these variables, but also the interactive effects between corona semanticity and age, time, and gender respectively, because we were interested in examining whether Corona Semanticity, as a characteristic of children's narratives, may help explain the effects of age, gender and time on children's well‐being. We found that initially higher Corona Semanticity was associated with lower well‐being in 11–12‐year‐olds (*β* = −71.79, *t* = −2.95, *p* < .01) and 15‐ to 16 ‐year‐olds (*β* = −66.65, *t* = −2.74, *p* < .01) compared to 8–9 year‐olds. However, over time, greater Corona Semanticity actually predicted greater well‐being in 11‐ to 12‐year‐old females (*β* = 84.28, *t* = 3.10, *p* < .01) and 15‐ to 16 ‐year‐old females (*β* = 70.46, *t* = 2.60, *p* < .01).

Additionally, higher levels of Corona Semanticity (*β* = 0.42, *t* = 2.43, *p* < .05), predicted higher levels of depressive symptoms measured by the SMFQ over time. The model measuring depressive symptoms (SMFQ) and the interactive effects between Corona Semanticity and age, time, and gender showed that, over time, greater Corona Semanticity predicted less depressive symptoms in both 11‐ to 12‐year‐old females (*β* = −68.59, *t* = −2.63, *p* < .01) and 15‐ to 16‐year‐old females (*β* = −58.72, *t* = 2.26, *p* < .05) (a table of the full models on Corona Semanticity can be found in the Supporting Information). We tested a similar model on negativity with the interactive effects between negativity, age, time and gender to assess potential effects of age, gender and time on children's mental well‐being. The results indicated that greater negativity was associated with better well‐being in younger 8–9 year‐old females (*β* = 10.67, *t* = 2.88, *p* < .01). However, in both 11‐ to 12‐year‐old females (*β* = −43.42, *t* = −2.23, *p* < .05) and in 15‐ to 16‐year‐old females (*β* = −56.12, *t* = −3.01, *p* < .01) higher levels of negative content was associated with worse mental well‐being. Surprisingly, over time, greater emotional negative content predicted better mental well‐being in 11–12 year‐old females (*β* = 30.18, *t* = 2.93, *p* < .01) and in 15–16 year‐old females (*β* = 35.11, *t* = 3.38, *p* < .001).

## DISCUSSION

The lockdown periods in response to the COVID‐19 pandemic represented highly disruptive and unusual situations. In the present study, we sought to learn how children and adolescents remembered that experience and whether it related to their psychological adjustment. We therefore used a longitudinal approach to examine age‐ and gender‐related differences in autobiographical memories from the COVID‐19 lockdowns from June 2020 to June 2021 in 8‐ to 16‐year‐olds, as well as memory aspects that might influence psychological adjustment during the time period. We addressed these goals by assessing autobiographical memories, psychological well‐being, and depressive symptoms at three different time‐points.

We expected changes in autobiographical memory for the lockdown experience over time with an overall decrease in Episodicity and Corona Semanticity, and an overall increase in negative affect. We found that the older age groups, and especially adolescent females were more elaborative and more emotional in their narratives compared to younger children, which is in accordance with findings from previous studies (e.g., Fivush, 2012; Fivush & Baker‐Ward, 2005). Furthermore, the memories demonstrated a decrease in Episodicity and Corona Semanticity over time. Surprisingly, we also found a decrease in negative affect over time, suggesting that memories lost their emotional content along with other details. Consistent with this finding, our analysis reported in Supporting Information revealed that children and adolescents also rated their memories with decreasing intensity over the three assessment points. Our findings are consistent with Brown's predictions (2021) of how the transition from pre‐ to the post‐ COVID‐19‐life would affect people's memories. The change in people's lives during the lockdowns limited people's everyday lives, including imposing heavily enforced restrictions on leaving the house, activities outside the home, and opportunities to meet with others in person. Brown (2021) argued that the situation would have implications for both the organization and the contents of autobiographical memory, leading to both a ‘COVID bump’ and a ‘lockdown dip’. The results from the present study seem to support Brown's (2021) predictions. First, the decrease in Episodicity, Corona Semanticity and affect over time might reflect that the first lockdown—as a first‐time experience—was initially more memorable among the children and adolescents because of the novelty and distinctiveness experienced in the very beginning (Rubin et al., 1998; Thomsen & Berntsen, 2005). However, as children and adolescents during lockdowns were restricted from in‐person socialization and were unable to leave their residence over extended periods of time, their experiences became less unique and more schematized (e.g., Conrad et al., 1998; Neisser, 1981). Consequently, there were fewer opportunities to experience interesting and memorable events.

As predicted, our results also showed that children's and adolescents' mental health decreased over time and that adolescent females fared the worst at all time‐points. Our findings are in accordance with other studies assessing children's and adolescents' mental health during COVID‐19 lockdowns (e.g., Halldorsdottir, 2021; Meherali et al., 2021; Orben et al., 2020), with adolescent females generally exhibiting stronger declines in well‐being and mental health compared to adolescent males (Halldorsdottir, 2021; Houghton, 2022).

The present study, however, is unique in that it is, to the best of our knowledge, the first and only longitudinal study assessing whether the content of autobiographical memory predicts declines in psychological adjustment in children and adolescents during COVID‐19 lockdowns. Consistent with our predictions, we found that children and adolescents whose narratives yielded higher scores in negative affect and included more information about COVID‐19 and the resulting restrictions, fared the worst over time. A possible explanation might be that those who integrated more COVID‐19‐related information in their memories, may also have integrated COVID‐19 lockdown experiences more profoundly in the development and formation of their selves during the same period. McLean and colleagues (2007) emphasize that autobiographical memories typically provide a window into understanding how selves are created, which might be particularly essential during adolescence, where identity‐formation is undergoing critical developmental progress and where the integration of internal states into personal memories develops substantially (Fivush, 2012; Habermas & Bluck, 2000). In adolescence, autobiographical memories begin to form an overarching narrative of one's life, where specific recollections are typically integrated into more hierarchical structures (McLean & Pasupathi, 2009). Given the age range of 8‐ to 16‐ year‐olds participating in the present study, the memories from the lockdown might be integrated in their narratives to a different extent as a function of age. According to Habermas and de Silveria (2008), 8‐year‐olds are still not able to fully order their personal experiences around coherent, temporally organized, theme, despite their ability to remember the order of their experiences (e.g., Pathman et al., 2022; Pathman & Ghetti, 2014; Sipe & Pathman, 2021). In contrast, adolescents typically begin to provide such cohesive narrative of their previous experiences from around 12 years of age (Reese et al., 2010). Development during adolescence beyond 12 years of age brings changes in the content in the autobiographical memories in that they are more likely to include evaluations and possible casual connections between life events. Consequently, adolescent participants in the present study may better integrate their lockdown memories into their meaning‐making narratives to a higher degree compared to their younger peers. This may result in differences in the effects of autobiographical memories over time.

It is well established that autobiographical memories provide a foundation for emotional well‐being (e.g., Banks & Salmon, 2013; Fivush & Baker‐Ward, 2005). Thanks to our longitudinal design, not only did we find that autobiographical memory and emotional well‐being are correlated at any given time, but also that memory characteristics about the COVID‐19 predict well‐being longitudinally above and beyond concurrent relations. Indeed, we found that greater Corona Semanticity, but *not* Episodicity, was related to worse emotional well‐being over time. Most studies assessing relations between emotional well‐being and autobiographical memory, primarily in adults, show a tendency towards over‐general memory (i.e., memory with reduced Episodicity) in individuals who exhibit concurrent depressive symptoms (Watson & Berntsen, 2015). Our examination of Corona Semanticity in its own right, not merely the opposite of Episodicity, allowed us to distinguish decreases in episodic detail from increases in inclusion of semantic information. We found an increase in semantic corona‐related information integrated in the children's and adolescent's memories. Only few studies have examined both episodic and semantic autobiographical memory during late childhood and early adolescence (Willoughby et al., 2012), and longitudinal studies assessing relations between emotional well‐being and autobiographical memory content in childhood and adolescence are largely absent (but see Stange et al., 2013; Warne et al., 2020). Willoughby and colleagues (2012) studied both episodic and sematic autobiographical memory performance in 8‐ to 16‐year‐olds. They found that age‐related differences were stronger in Episodicity compared to semanticity and that females overall recalled more episodic details, but no more semantic details, than males.

In our study, we replicated age‐related differences in Episodicity consistent with the idea that older compared to younger children include more vivid detail in their recount about their past. This trend is mirrored in subjective assessments of clarity (available in the Supporting Information). Intriguingly, however, our separate coding of Episodicity and semanticity revealed that both of these components vaned over time, but that those children who included greater semanticity in their recounts showed greater decline in psychological well‐being over time and more depressive symptoms. One possible, but speculative explanation might be, that adolescents who ruminate more on semantic aspects of COVID‐19 perhaps have a tendency to distance themselves from the more personal content in the memories. Research has suggested that remembered memories may remain of more general character if the conceptual information that is activated during the early stages of retrieval is related to one's self‐representation (Williams et al., 2007). Moreover, recollections of nonspecific memories may serve as a functional avoidance short‐term strategy to reduce emotional discomfort associated with past experiences, whereas it may have negative long‐term impact on for instance problem‐solving strategies and affect regulation (Williams, 2006).

We recognize several limitations of the present study. First, one of the assessments of well‐being and depressive symptoms at the Time 1 was retrospective as thus dependent on the participants' ability to remembering their well‐being. We did our best to circumvent this challenge by planning and conducting the Time 1 Assessment immediately after re‐opening following the first lockdown. Thus, the retention time between lockdown and assessment was scheduled to a minimum. Second, younger children might have found the task more difficult to complete compared to their older peers. Although this aspect of the methods may inflate the correlation between memory and assessment of well‐being on the first assessment because they both rely on memory for the lockdown, this was not the case for the subsequent assessments when participants were asked to remember the past but assess their current psychological well‐being thereby contributing data for our longitudinal models that was unencumbered by this limitation. Moreover, our measures of well‐being and depressive symptoms exhibited high levels of reliability in all age groups, and the analyses on memory content reported in the main manuscript did not rely on participants' self‐report, but on coders' reliable ratings. These facts provide some reassurance about the quality of our data. Second, our data collection procedures were slightly different for Time 1 and Time 3 (when a member of our team was physically present during data collection at school after providing extensive explanations) and Time 2 (when teachers overshow data collection during remote instruction). Prior to data collection at Time 2, the teachers received written instructions about how to assist their students appropriately. Third, the present study was conducted with Danish children and adolescents. According to Cole et al. (2023) and Öner et al. (2023) the content in adults‘ memory recollections during the pandemic was affected by the stringency of governmental response and by the severity of the pandemic on a national level. Thus, we cannot rule out the possibility that the content in children's and adolescents’ memories from children raised in different countries and communities would differ in their phenomenological characteristics compared to a Danish population. Still, the present study provides important insights into age and gender differences and how the content in the memories predicted psychological adjustment in this unusual period of time. Finally, we experienced loss of participants at the follow‐ups. Nonetheless, the analyses were conducted by using multilevel modeling which is a robust method to missing data and sensitive to differences in groups (Bryk & Raudenbush, 1987; Tasca & Gallop, 2009). Moreover, the analyses to compare differences in retained versus lost participants showed no differences in either gender, age, WHO‐5 scores or SMFQ scores. Thus, the present study provides important insights into children's and adolescents' autobiographical memories and psychological adjustment in times of uncertainty and lockdowns.

## CONCLUSION

Together, the results provide novel and essential insights into children's and adolescents' experiences of the COVID‐19‐related lockdowns. Analyses assessing the relation between autobiographical memory and psychological adjustment suggest that thinking back on the personal past during the COVID‐19 lockdowns with more factual information about the lockdown itself and with more negative affect, negatively impacted psychological adjustment over time and when children returned to school. Future work might benefit from investigating how both semantic and episodic aspects of specific past experiences might be linked to children's and adolescents' psychological adjustment and coping in difficult times. In addition, future research should examine how memory narratives concerning challenging times, such as the lockdowns, might be different from other types of children's narratives, including children's and adolescents' personal memories about other relevant events and projections to the future.

### Sociocultural policy statement and statement on confirmatory versus exploratory research

We confirm, that the present study conforms with the SRCD sociocultural policy requirements. The study reported here represents a mixture of confirmatory and exploratory research. While we had specific hypotheses regarding changes in mental health and coded memory content over time, we did not make strong predictions about memory themes or memory self‐ratings.

## Supporting information


Data S1.

## Data Availability

Data, code, and materials are available from the first author upon reasonable request. The analyses presented here were not preregistered.
