# Supplementary material for: Remembering history: Autobiographical memory for the COVID‐19 pandemic lockdowns, psychological adjustment, and their relation over time
Source: Child Dev. 2024 Aug 14;96(1):55–70. doi: 10.1111/cdev.14131 (PMC11693838; doi:10.1111/cdev.14131)
Supplement: Supplementary file 1 — Data S1. [file CDEV-96-55-s001.zip › MemoryThemes_SuppInfo.docx]

**Supplemental information: Memory Themes**

***Theme.*** The main theme of each memory was coded by three different raters. First, rater ‘G’ identified a list of themes by reading thorough all the memories. A total number of 38 themes of the lockdown memories were identified. Subsequently, a number of 22 of the overall themes were identified as involving content of previous or current ‘social life’. A second rater ‘H’ and a third rater ‘I’ coded 20 % of the memories. Interrater agreement between them was acceptable 82.9%, κ = .808. Disagreements between rater ‘H’ and ‘I’ were solved by discussion. Finally, rater ‘C’ coded the remaining 80% of the memories. Rater ‘I’s codings were used in the analyses.

| **Theme** | **Time 1** | | **Time 2** | | **Time 3** | | |
| --- | --- | --- | --- | --- | --- | --- | --- |
|  | Frequency | Percentage | Frequency | Percentage | Frequency | Percentage | |
| Seeing friends | 28 | 11.4 | 18 | 11.9 | 13 | | 6.6 |
| Announcement of the lockdown | 35 | 14.2 | 9 | 6.0 | 23 | | 11.7 |
| A trip | 13 | 5.3 | 14 | 9.3 | 8 | | 4.1 |
| Seeing grandparents | 11 | 4.5 | - | - | 3 | | 4.1 |
| Visiting a weekend cottage | 6 | 2.4 | 7 | 4.6 | 7 | | 3.6 |
| Gaming | 20 | 8.1 | 5 | 3.3 | 6 | | 3.1 |
| Virtual lectures and meetings | 11 | 4.5 | - | - | - | | - |
| Home-school | 2 | 0.8 | 11 | 7.3 | 16 | | 8.2 |
| Going for a walk | 9 | 3.6 | 4 | 2.6 | - | | - |
| Family qualitytime | 13 | 5.3 | 6 | 4.0 | 9 | | 4.6 |
| Outside ballgames | 4 | 1.6 | - | - | 1 | | 0.5 |
| Birthday and party | 6 | 2.4 | 6 | 4.0 | 8 | | 4.1 |
| Everyday life | 13 | 5.3 | 5 | 3.3 | 3 | | 1.5 |
| COVID-19 guidelines | 3 | 1.2 | 1 | 0.7 | 2 | | 1.0 |
| Buying something new | 3 | 1.2 | 2 | 1.3 | 4 | | 2.0 |
| Digital ’come-together’ | 1 | 0.4 | 2 | 1.3 | 2 | | 1.0 |
| Quarrels with friends/family | 3 | 1.2 | 1 | 0.7 | 3 | | 1.5 |
| Partners/relatipnships | 4 | 1.6 | 1 | 0.7 | 1 | | 0.5 |
| Trampoline | 3 | 1.2 | - | - | 1 | | 0.5 |
| Playing outside | - | - | 5 | 3.3 | 4 | | 2.0 |
| COVID-19 tests | 2 | 0.8 | 3 | 2.0 | 3 | | 1.5 |
| Physical exercise | 3 | 1.2 | 8 | 5.3 | 5 | | 2.6 |
| Pets | 9 | 3.7 | 4 | 2.6 | 3 | | 1.5 |
| Learning something new | 3 | 1.2 | - | - | - | | - |
| Missing someone | 4 | 1.6 | 3 | 2.0 | 2 | | 1.0 |
| Announcement of re-opening | 1 | 0.4- | 1 | 0.7 | 3 | | 1.5 |
| Watching TV | 3 | 1.2 | 2 | 1.3 | 2 | | 1.0 |
| Illness/death among loved ones | 4 | 1.6 | 1 | 0.7 | 5 | | 2.6 |
| Boredom | 4 | 1.6 | 1 | 0.7 | 7 | | 3.6 |
| Crying | 1 | 0.4 | - | - | 1 | | 0.5 |
| Stress/ anxiety/ worry/ sadness | - | - | 5 | 3.3 | 7 | | 3.6 |
| Loneliness | 3 | 1.2 | 2 | 1.3 | - | | - |
| Cancelled events | 3 | 1.2 | 4 | 2.6 | 5 | | 2.6 |
| Cooking/ baking | 3 | 1.2 | - | - | 3 | | 1.5 |
| Ramadan | 1 | 0.4 | - | - | - | | - |
| Christmas | - | - | 8 | 5.3 | 1 | | 0.5 |
| New Year’s Eve | - | - | 4 | 2.6 | - | | - |
| ‘Can’t think of any memory’ | - | - | 8 | 5.3 | 33 | | 16.8 |
| Other | 13 | 5.3 | - | - | 1 | | 0.5 |

*Themes – Descriptive Results*

Themes were assessed to examine a potential decrease in frequency of certain themes over time and to assess age differences. We found memories concerning current or previous social life across all ages. For instance, a 15-year-old boy wrote: “I’m at home playing at my computer. I feel a bit lonely sometimes. I just miss a normal school day meeting with my friends.” The social content in the memories over time showed a frequency of 33.3 % at Time 1, 36.6% at Time 2, and at Time 3 19.4%. Interestingly, at the two first time points only 12- and 15-year-olds reminisced about the moment when they first learned about the lockdown. For instance, a 15-year-old girl wrote: "“It was a Wednesday night and my grandmother was visiting to cook. I had just come home from practice and everyone was sitting in front of the TV, that is, my sister, my mother and my grandmother. Mette Frederiksen (the Danish Prime Minister) was about to speak. It was a huge shock. She announced that we should not go to school tomorrow, or the following weeks. Even my sister and my mother had to stay at home. It felt like my whole world just fell apart. I had to go to the bathroom, just to do something that was totally normal.” At Time 1, 21.9 % of the 11-year-olds’ memories and 18.6 % of the 15-year-olds’ memories addressed the announcement of the lockdown, and at Time 2, 11.3 % of the 12-year-olds’ and 4.1% of the 15-year-olds’ memories were about when they first heard the announcement of the lockdown. At Time 3, a small proportion (3.8%) of the 9-year-olds reported to remember when they first learned about the lockdown, whereas the frequency was 18.8% in the 12-year-olds and 11.4% in the 16-year-olds. At Time 2 and Time 3 a new category, not presented at Time 1, was identified which was the theme “Can’t think of any memory”. At Time 2, 16.7% of the 9-year-olds, 3.8% of the 12-year-olds, and 2.7% of the 16-year-olds reported that they were unable to think of any specific memory. At Time 3, this tendency increased even more, so that 20.8% of the 9-year-olds, 10.9% of the 12-year-olds, and 19% of the 16-year-olds reported that they could not think of a memory. For instance, a 16-year-old girl wrote “I can’t remember anything specific that happened, ‘cause every day was just alike”, and a 16-year-old boy reported “I don’t make memories just sitting in front of my computer all day long.”
