# Supplementary material for: Remembering history: Autobiographical memory for the COVID‐19 pandemic lockdowns, psychological adjustment, and their relation over time
Source: Child Dev. 2024 Aug 14;96(1):55–70. doi: 10.1111/cdev.14131 (PMC11693838; doi:10.1111/cdev.14131)
Supplement: Supplementary file 1 — Data S1. [file CDEV-96-55-s001.zip › Models_SuppInfo.docx]

**Model predicting well-being from Corona Semanticity**

| **Model** | **Estimate** | **St error** | **t-value** | **p-value** |
| --- | --- | --- | --- | --- |
| (Intercept) | 684.096 | 4.511 | 15.165 | 2e-16*** |
| CoronaSemanticity | 68.461 | 24.023 | 2.85 | < .01** |
| Time | -35.304 | 26.179 | -1.349 | 0.17826 |
| Female | -79.475 | 63.244 | -1.257 | 0.20947 |
| 11-12-year-olds | 50.347 | 67.886 | 0.742 | 0.45866 |
| 15-16-year-olds | 51.854 | 63.667 | 0.814 | 0.41577 |
| CoronaSemanticity:Time | -32.082 | 109.288 | -2.936 | < .01** |
| CoronaSemanticity:Female | -778.451 | 266.206 | -2.924 | < .01** |
| Time:Female | 81.416 | 35.987 | 2.262 | < .05* |
| CoronaSemanticity:11-12 year-olds | -717.884 | 243.679 | -2.946 | < .01** |
| CoronaSemanticity:15-16 year-olds | -666.545 | 243.585 | -2.736 | < .01** |
| Time:11-12 year-olds | -0.4192 | 34.348 | -0.122 | 0.90293 |
| Time:15-16 year-olds | 20.984 | 3.312 | 0.634 | 0.52675 |
| Female:11-12 year-olds | 23.484 | 94.141 | 0.249 | 0.80311 |
| Female:15-16 year-olds | 42.148 | 90.731 | 0.465 | 0.64246 |
| CoronaSemanticity:Time:Female | 302.629 | 118.185 | 2.561 | < .05* |
| CoronaSemanticity:Time:11-12 year-olds | 360.049 | 111.634 | 3.225 | < .01** |
| CoronaSemanticity:Time:15-16 year-olds | 302.422 | 111.507 | 2.712 | < .01** |
| CoronaSemanticity:Female:11-12 year-olds | 842.776 | 272.339 | 3.095 | < .01** |
| CoronaSemanticity:Female:15-16 year-olds | 704.645 | 271.221 | 2.598 | < .01** |
| Time:Female:11-12 year-olds | -7.598 | 47.162 | -1.611 | 0.10807 |
| Time:Female:15-16 year-olds | -144.015 | 45.559 | -3.161 | < .01** |
| CoronaSemanticity:Time:Female:11-12 year-olds | -350.616 | 122.051 | -2.873 | < .01** |
| CoronaSemanticity:Time:Female:15-16 year-olds | -253.177 | 121.521 | -2.083 | < .05* |

*Note.* Model predicting well-being (WHO-5 scores) from Corona Semanticity with the interactions with time, age and gender.

**Model predicting depressive symptoms from Corona Semanticity**

| **Model** | **Estimate** | **St error** | **t-value** | **p-value** |
| --- | --- | --- | --- | --- |
| (Intercept) | 90.554 | 10.831 | 8.361 | 6.34E-16*** |
| CoronaSemanticity | -14.773 | 56.093 | -2.634 | < .001 |
| Time | -27.854 | 15.181 | -1.835 | 0.067143 |
| Female | -38.106 | 16.689 | -2.283 | < .05* |
| 11-12-year-olds | -54.919 | 15.394 | -3.568 | < .001*** |
| 15-16-year-olds | -11.795 | 0.6144 | -1.92 | 0.055661 |
| CoronaSemanticity:Time | 158.389 | 62.591 | 2.531 | < .05* |
| CoronaSemanticity:Female | 144.482 | 5.697 | 2.536 | < .05* |
| Time:Female | 134.035 | 56.909 | 2.355 | < .05* |
| CoronaSemanticity:11-12 year-olds | 35.886 | 23.042 | 1.557 | 0.120021 |
| CoronaSemanticity:15-16 year-olds | 33.862 | 21.831 | 1.551 | 0.121521 |
| Time:11-12 year-olds | 68.691 | 25.514 | 2.692 | < .001** |
| Time:15-16 year-olds | 0.5165 | 0.8436 | 0.612 | 0.540708 |
| Female:11-12 year-olds | 0.9542 | 0.8472 | 1.126 | 0.260819 |
| Female:15-16 year-olds | 0.9724 | 0.7772 | 1.251 | 0.211686 |
| CoronaSemanticity:Time:Female | -165.494 | 64.319 | -2.573 | < .05* |
| CoronaSemanticity:Time:11-12 year-olds | -132.597 | 63.782 | -2.079 | < . 05* |
| CoronaSemanticity:Time:15-16 year-olds | -71.164 | 27.712 | -2.568 | < .05* |
| CoronaSemanticity:Female:11-12 year-olds | -68.589 | 26.128 | -2.625 | < .01** |
| CoronaSemanticity:Female:15-16 year-olds | -58.721 | 26.039 | -2.255 | < .05* |
| Time:Female:11-12 year-olds | 0.1851 | 11.497 | 0.161 | 0.872198 |
| Time:Female:15-16 year-olds | 0.9055 | 10.669 | 0.849 | 0.39662 |
| CoronaSemanticity:Time:Female:11-12 year-olds | 74.649 | 28.898 | 2.583 | < .05* |
| CoronaSemanticity:Time:Female:15-16 year-olds | 54.547 | 28.491 | 1.915 | 0.056338 |

*Note.* Model predicting depressive symptoms (SMFQ scores) from Corona Semanticity with the interactions with time, age and gender.
