# Supplementary material for: Remembering history: Autobiographical memory for the COVID‐19 pandemic lockdowns, psychological adjustment, and their relation over time
Source: Child Dev. 2024 Aug 14;96(1):55–70. doi: 10.1111/cdev.14131 (PMC11693838; doi:10.1111/cdev.14131)

**Supplemental information: Subjective memory ratings**

# Methods on subjective memory ratings

The participants were asked to describe the most memorable personal event from the lockdown period. Instructions emphasized that there was no right or wrong answers and that we were interested in their own personal experiences from the period. Specifically, the instructions state: “Please write down as many details as possible about a memory from your personal life during the COVID-19 lockdown.” After providing their narrative, participants were asked to rate several aspects of their memory using Likert scales ranging from 1 = not at all to 5 = Very much. Specifically, participants assessed the extent to which their memory for the event was: (a) Positive; (b) Negative; (c) Important to me; (d) Stressful; (e) “Hard to think about”. In addition, using a 5-point Likert scale, they answered the questions: “How well do you remember this life event?” as (a) Clearly (from not clearly at all to very clearly); (b) Detailed (from not detailed at all to very detailed); and “How does the memory make you feel?” (all emotions rated from ‘not at all’ to ‘very much’): (a) Happy; (b) Satisfied; (c) Calm; (d) Sad; (e) Angry; (f) Worried; and “How often have you thought about this memory?” (from never to very often), and (V) “How often have you talked about this memory?” (from never to very often). Finally, participants were asked to report whether they could see the event primarily from a first person perspective (‘I’) or a third person perspective (‘he’/’she’) if they could visualize the specific personal event in their mind.

**Results on subjective memory ratings**

Factor analyses were conducted to assess interrelations between the different autobiographical memory Likert scale items. The factor analyses revealed that the 4 items ‘positive’, ‘happy’, ‘satisfied’ and ‘calm’ were highly interrelated, α = .91, and the 5 items ‘negative’, ‘stressful’, ‘angry’, ‘sad’ and ‘worried’ were highly interrelated, α = .85. Therefore, we used composite scores for analyses concerning Positive and Negative valence. In addition, we used self-rating measures 1) Importance; 2) Hard to think about; 3) Clearly remembering; 4) Detailed remembering; 5) Occurrence of thinking about the memory; and 6) Frequency of talking about the memory.

*Subjective measures over time*

Multilevel modelling was conducted to assess changes in the subjectively rated content of the memories as a function of time, age and gender. The figures below illustrate our findings:


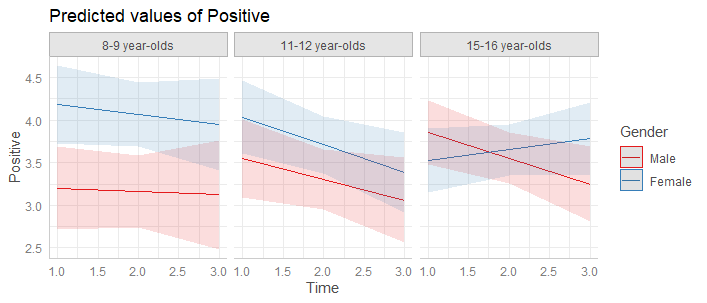


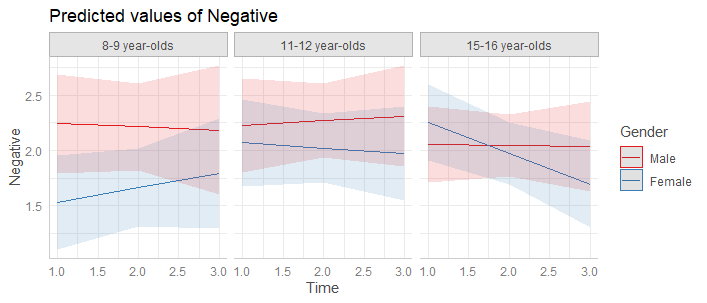


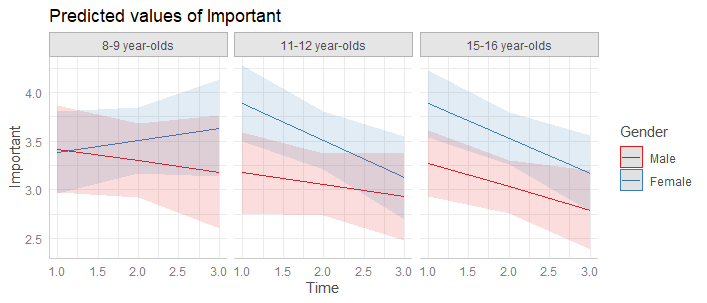


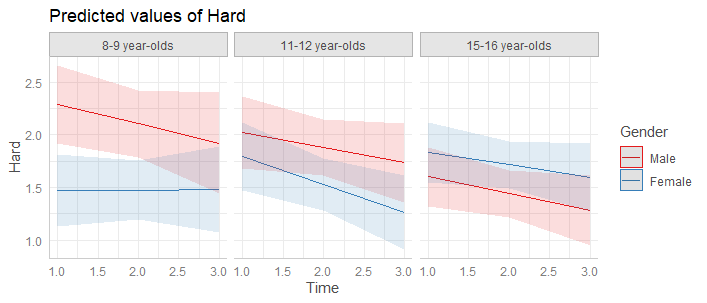


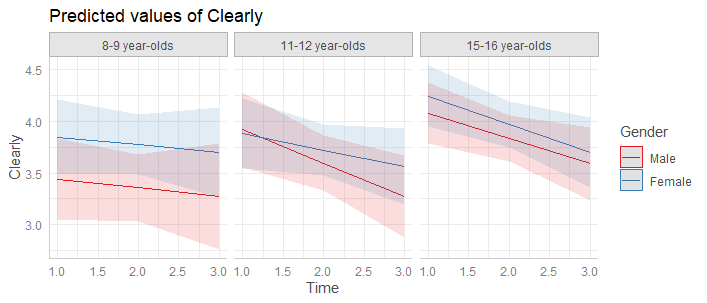


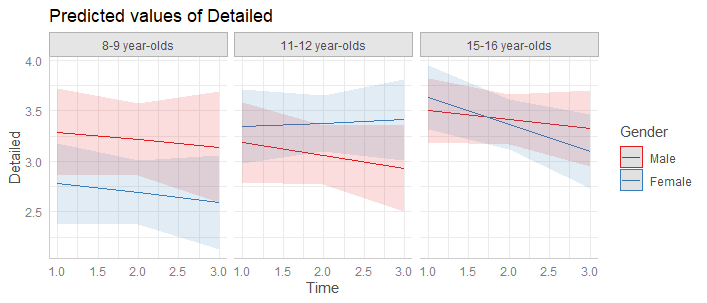


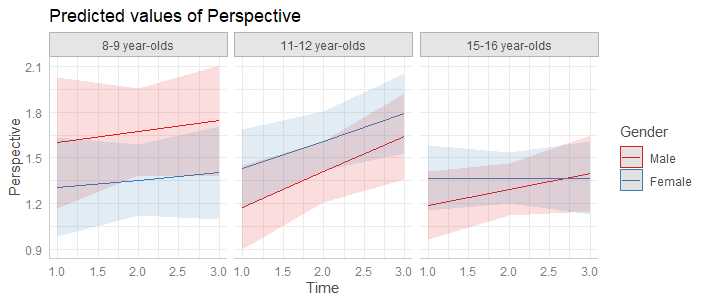


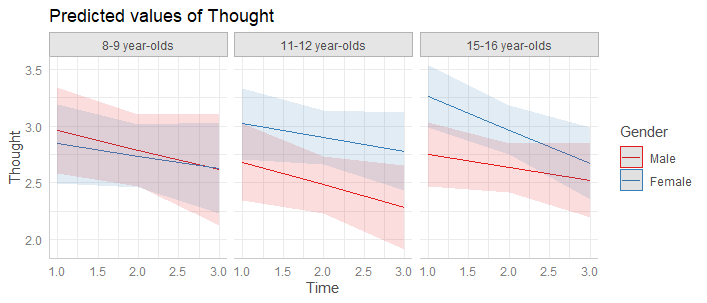


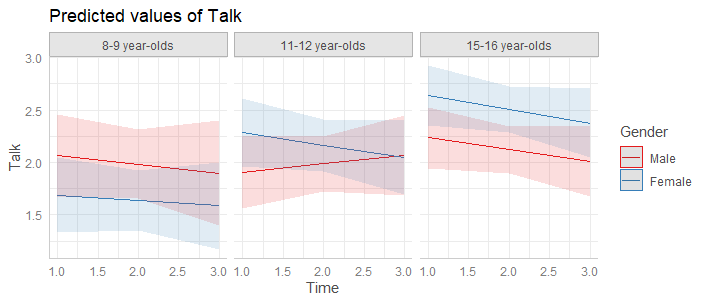


## Subjective memory ratings predicting psychological adjustment

We tested the relation between the content of memories and psychological adjustment. By applying multilevel modeling, we first modeled psychological adjustment over time while accounting for gender and age. For the current results, to the previous model we added subjective ratings of the memories as predictors with an interaction of time to analyze the trajectory of these relationships. The tables below display our findings:


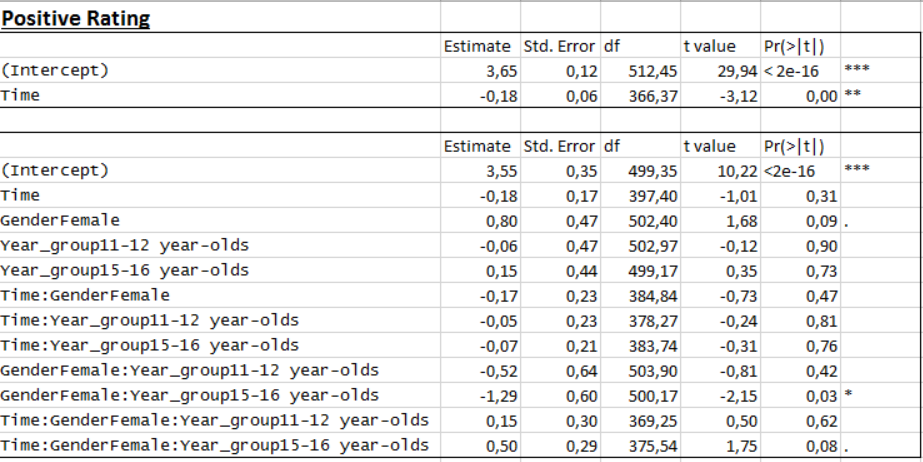


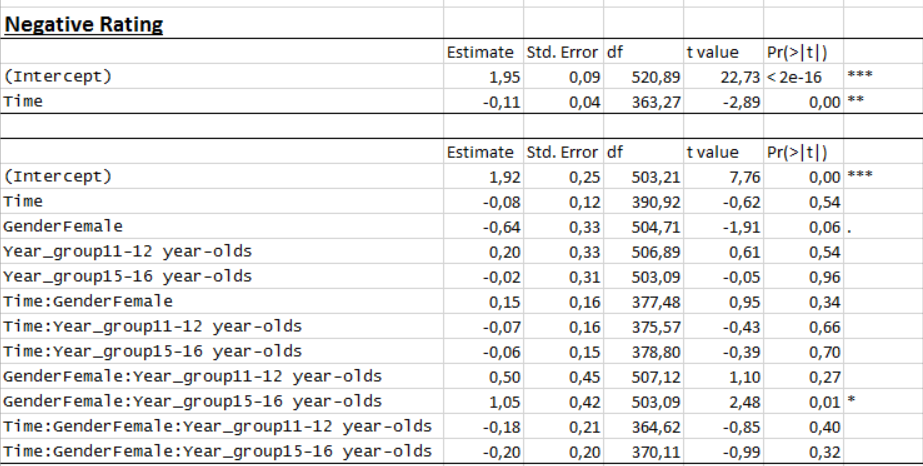


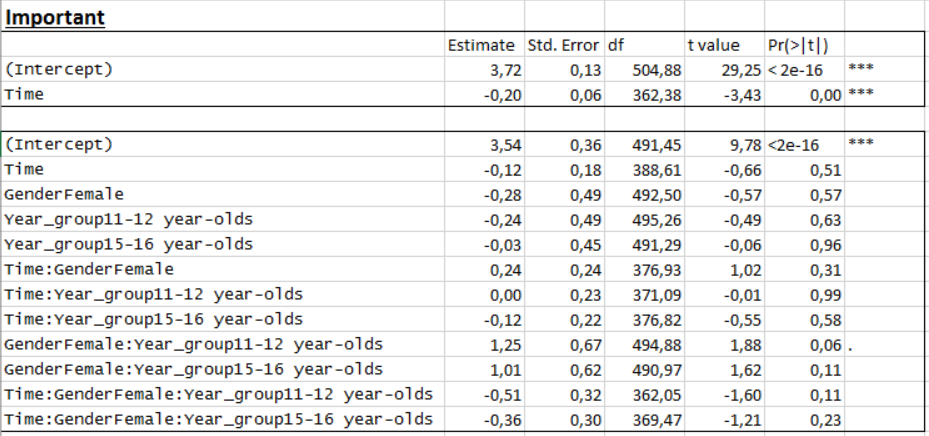


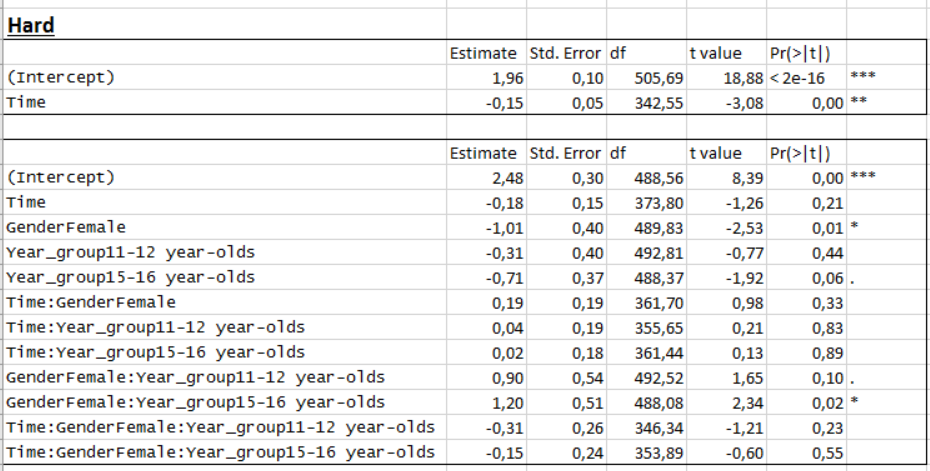


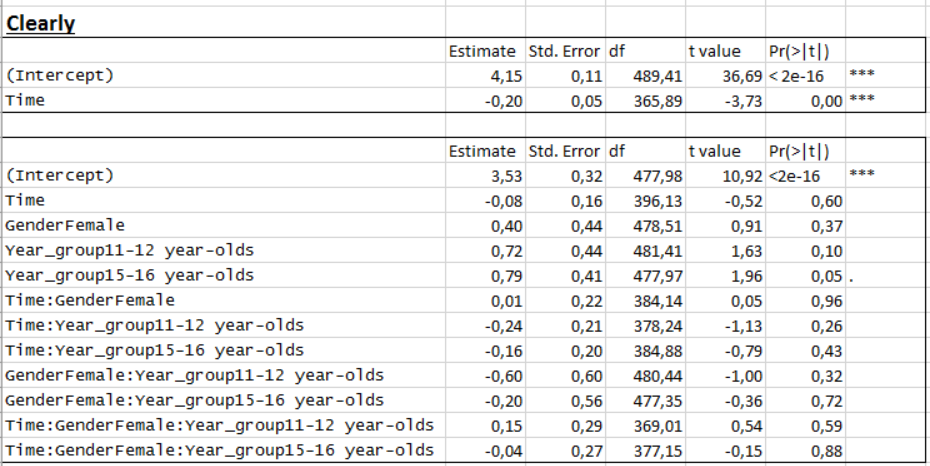


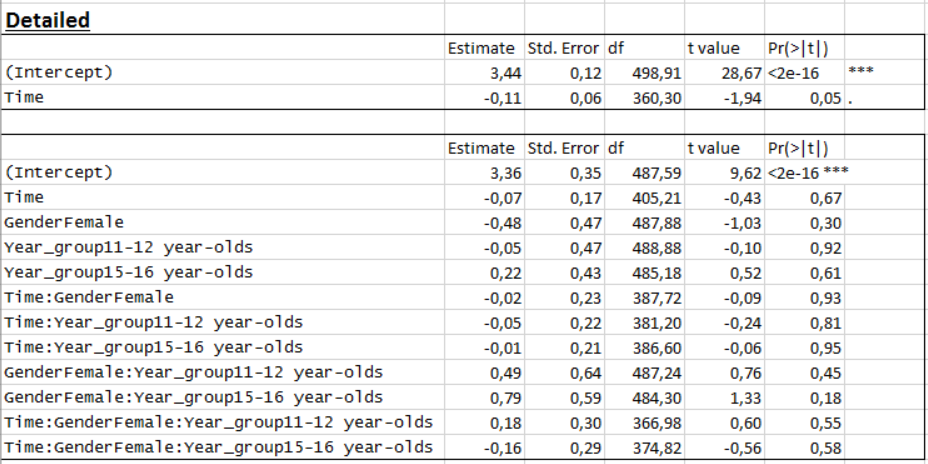


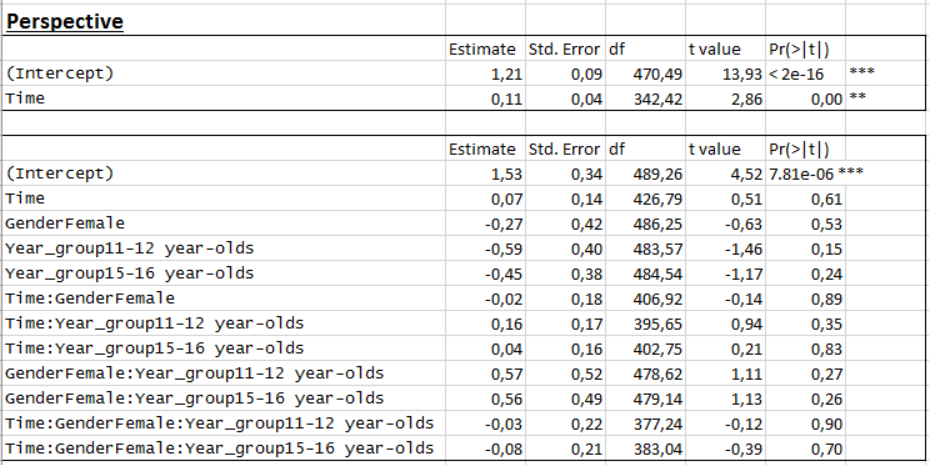


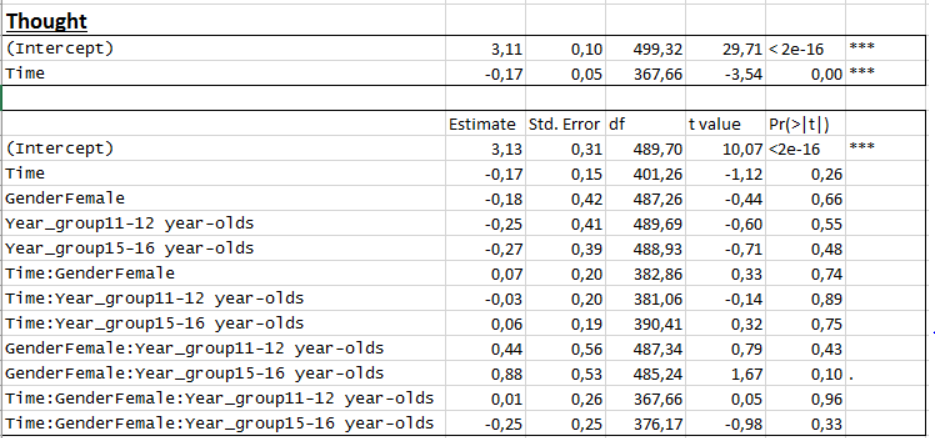


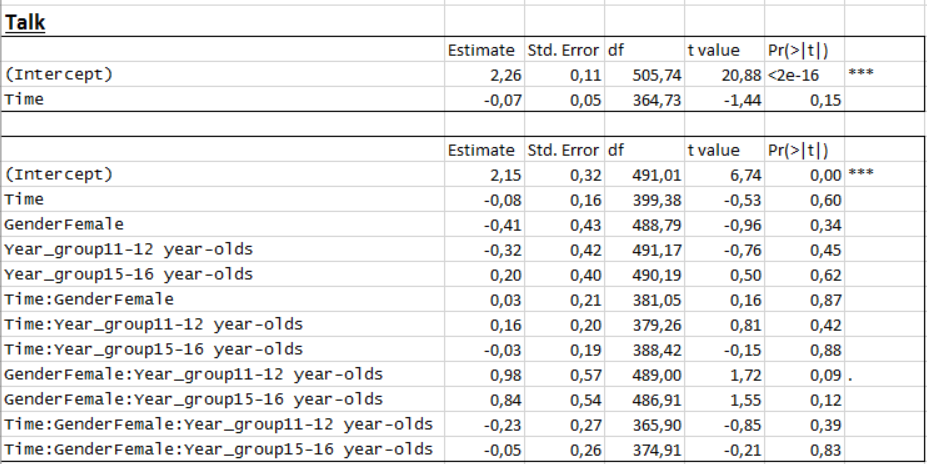

Supplement: Supplementary file 1 — Data S1. [file CDEV-96-55-s001.zip › SubjectiveRatings_SuppInfo.docx]
